# What is a “Distinctive Nutritional Requirement”? A Position Paper of the Healthcare Nutrition Council^☆^

**DOI:** 10.1016/j.cdnut.2023.102013

**Published:** 2023-10-07

**Authors:** Katie Chmielecki, Berit Dockter, Wendy Johnson, Madeline Jurch, Pamela Pacht, Jena Rostorfer

**Affiliations:** 1 Abbott Nutrition, Columbus, OH, United States; 2 Healthcare Nutrition Council, Washington, DC, United States; 3 Nestlé USA (retired), Arlington, VA, United States; 4 Nutricia North America, Rockville, MD, United States; 5 Market Access, Nestlé Health Science, Bridgewater, NY, United States; 6 Abbott Nutrition, Columbus, OH, United States

**Keywords:** distinctive nutritional requirements, enteral nutrition, foods for special dietary use, medical foods, nutritional supplements, oral nutritional supplements

## Abstract

The Healthcare Nutrition Council (HNC) represents manufacturers of enteral nutrition formulas and oral nutrition supplements, including those categorized as medical foods and parenteral nutrition. HNC member companies, Abbott’s Nutrition Division, Nestlé Health Science, and Nutricia North America, a subsidiary of Danone S.A., manufacture a majority of the medical foods consumed in the United States. HNC is proposing a modernized interpretation of the medical food framework to reflect the evolution of nutrition science and health care. The medical food category was first defined in 1988 as part of the Orphan Drug Act. Since then, the scientific community's understanding of nutrition and the role it can play in disease management has progressed. HNC believes that a patient-centric approach is needed to foster research and innovation and to position medical foods as a viable solution in the dietary management of disease. HNC proposes that *distinctive nutritional requirements* refer to the clinical need for a specific nutritional intake (compared with the intake of healthy populations), which may exist by reason of abnormal physiologic manifestation or physical impairment associated with a disease or condition. The dietary management of these diseases and conditions results in clinically meaningful improvements, including but not limited to nutritional status, health outcomes, or quality of life. HNC believes that abnormal physiologic manifestation or physical impairment would include a limited, impaired, or disturbed capacity to ingest, digest, absorb, metabolize, or excrete ordinary food or certain nutrients or metabolites or other medically determined requirements for nutrients or other food substances of biological value. HNC recommends our position be considered as we build consensus across the industry. We request that the Food and Drug Administration modify and codify the current definition to reflect this. Patients and the health care system will benefit from a strong regulatory interpretation of the medical foods framework.

## Introduction

The Healthcare Nutrition Council (HNC) comprises manufacturers of enteral nutrition formulas and oral nutrition supplements, including those categorized as medical foods and parenteral nutrition. HNC member companies (Abbott’s Nutrition Division, Nestlé Health Science, and Nutricia North America, a subsidiary of Danone S.A.) manufacture a majority of the medical foods consumed in the United States and have decades of experience in safely and efficaciously meeting patients’ nutritional needs. The HNC strives to work with the US Food and Drug Administration (FDA) and other stakeholders to improve patient outcomes by advancing nutrition policies and actions that raise awareness and optimize access to essential nutrition support therapies.

In August 2019, HNC, in partnership with the American Society for Nutrition, held a workshop entitled the “Medical Foods Workshop: Science, Regulation, and Practical Aspects” [1] (the proceedings of which were published in this journal [2]). The objectives of the workshop were to advance dialogue on the scientific and regulatory status of medical foods in the United States, drive consensus on terms and definitions for medical foods, and ultimately help to improve patient access to these important products. The workshop included a variety of stakeholders, encompassing patients, clinicians, government officials, and the medical food industry.

In this paper, HNC furthers its efforts by proposing a modernized interpretation of the medical food framework to reflect the evolution of nutrition science and health care. The medical food category was first defined in 1988 as part of the Orphan Drug Act (ODA). Since then, the scientific community's understanding of nutrition and the role it can play in disease management has progressed, presenting new ways to optimize patient care. Conversely, the 1988 statutory definition of medical foods and the regulatory criteria that were later established through the 1993 Nutrition Labeling and Education Act (NLEA) do not reflect scientific advances. HNC believes a patient-centric approach is needed to foster research and innovation to position medical foods as a viable solution in the dietary management of disease. More specifically, HNC believes “distinctive nutritional requirements” (DNR), a critical term in the medical food statutory definition that serves as a fundamental prerequisite to the medical food category, should be revisited and updated through a more modern lens.

In the following sections, we

• provide historical context for our thinking,
• present our modernized interpretation of DNR, and
• summarize our recommendations for modernizing the regulatory framework for medical foods.

HNC is advocating for the use of our proposed position to build consensus across the industry to help ensure that people living with disease have access to medical foods not only to live but also to live better.

### Historical Context of Medical Foods

*Medical food* was defined in 1988 as part of the ODA amendments, thus creating a category distinct from the broader category of foods for special dietary use (for more historical context, we refer the reader to the summary of Tim Morck’s presentation at the Medical Foods Workshop [1]). (We share additional thoughts on the distinction between foods for special dietary use and medical foods in the **Supplementary Text Appendix.**) In 1988 a medical food was defined as [3]:

…a food which is formulated to be consumed or administered enterally under the supervision of a physician and which is intended for the specific dietary management of a disease or condition for which *distinctive nutritional requirements* [emphasis added], based on recognized scientific principles, are established by medical evaluation.

The FDA’s interpretation of the statute narrowly defines medical foods and constrains the types of products that fit within this category of food. In particular, the phrase “distinctive nutritional requirements” has been the subject of much debate and scientific exploration. Determining whether a medical food can be developed and marketed is dependent on whether a disease or condition results in a DNR or perhaps whether a DNR is the consequence of a disease or condition. To date, the FDA has not explicitly defined “distinctive nutritional requirements.”

As part of the 1990 NLEA, Congress incorporated the definition of medical food into the Federal Food Drug and Cosmetic Act. In 1993, the rules for implementing the NLEA codified the medical foods definition and established a means for such products to claim exemptions from conventional food nutrition labeling, provided that established criteria were met [4]. Despite the intent to claim labeling exemptions, the FDA has often used the established regulatory criteria as a means to further define and narrow the definition of the medical foods category and has especially used a portion of clause (ii) to narrowly constrain the definition of DNR: “…the dietary management of which cannot be achieved by the modification of the normal diet alone.” This clause, often referred to as modification of diet alone (“MODA”), has been the subject of much debate.

In 1996, the FDA published an advanced notice of proposed rulemaking (ANPRM) to further elaborate on the medical food regulatory framework [5]. In this ANPRM, the FDA proposed 2 interpretations of DNR for consideration: a physiologic interpretation and an alternative interpretation (Table 1).

**TABLE 1:** Existing and proposed interpretations of distinctive nutrient requirements

A Physiologic Interpretation of DNR by the FDA^a^	An Alternative Interpretation of DNR by the FDA^a^	A Modernized Interpretation of DNR by the Healthcare Nutrition Council
“[T]he body’s requirement for specific amounts of nutrients to maintain homeostasis (the state of equilibrium in the body with respect to various functions and to the chemical compositions of the fluids and tissues) and sustain life; that is, the amount of each nutrient that must be available for use in the metabolic and physiological processes necessary to sustain life. …the distinctive nutritional needs associated with a disease reflect the total amount needed by a healthy person to support life or maintain homeostasis, adjusted for the distinctive changes in the nutritional needs of the patient as a result of the effects of the disease process on absorption, metabolism, and extraction. These distinctive nutritional requirements may be greater than, less than, or in a narrower range of tolerance than for an otherwise healthy individual.”	“…those requirements that result from a disease or condition that cause a physical or physiological limitation in the ability of a person to ingest or digest conventional sources and result in that person needing specially formulated foods to meet part or all of their daily nutrients needs.”	The clinical need for a specific nutritional intake (compared with the intake of healthy populations) which may exist by reason of abnormal physiologic manifestation or physical impairment associated with a disease or condition, the dietary management of which results in clinically meaningful improvements, including but not limited to nutritional status, health outcomes, or quality of life.

Abbreviations: DNR, distinctive nutritional requirements; FDA, Food and Drug Administration.

a As proposed in Federal Register Doc No.: 96-30441 [5].

The alternative interpretation recognizes a “physical or physiological limitation in the ability of a person to ingest or digest conventional nutrient sources and result in that person needing specially formulated foods to meet part or all of their daily nutrients needs.” This would include someone whose life-support is a standard formula tube feeding because it could be a purely physiologic need. In their discussion of the alternative interpretation, the Agency stated [5],

*‘Distinctive nutritional requirement’ may also be interpreted to encompass physical or physiological limitations in a person’s ability to ingest or digest conventional foods, as well as distinctive physiological nutrient requirements.*

*…This definition would include uses that a purely physiological definition of ‘distinctive nutritional requirements’ would exclude, such as foods intended for persons not able to ingest foods in certain physical forms (e.g., solid food), foods intended for persons who need a concentrated form of nutrition because of reduced appetite as a result of disease or convalescence), or foods intended for persons who may have other physical limitations on the amount or composition of food that they can consume. Although these types of conditions do not necessarily result in nutrient needs different from those of healthy persons, they represent a situation where it may be necessary that the food be formulated and manufactured within very narrow tolerances to ensure that the food provides most or all of the essential nutrients, as the persons for whom the food is intended may not be able to eat a variety of foods to ensure that they meet their nutrient requirements.*

Although the ANPRM was withdrawn for administrative reasons in 2004, FDA has continued to reiterate that their opinions expressed within the ANPRM remain unchanged.

In 2007, FDA published a draft medical food guidance in the form of a question-and-answer document. A revised draft of the guidance included a specific class of diseases purported by FDA to appropriately necessitate a medical food under the statutory definition and regulatory criteria (e.g., inborn errors of metabolism) as well as indications purported by FDA to *not* meet their interpretation of the medical food criteria (e.g., diabetes and pregnancy). The revised language in the final guidance, published in 2016, states that “there are no distinctive nutritional requirements” associated with pregnancy or with the management of diabetes [6]. The final guidance implies that a nutritional requirement is “distinct” only if it cannot be met through modification of the normal diet alone [2]. This guidance does not, however, opine on the spectrum of severity of disease, management of comorbidities that may also present with compounding distinctive nutritional requirements, or patient feasibility of modifying a whole-food diet without assistance of medical food. The next section elucidates HNC’s thinking on DNR as it pertains to medical food research [[7], [8], [9]], innovation, and development.

## A Modernized Interpretation of Medical Foods

### A patient-centric approach

There has been little clarity on the FDA’s interpretation of DNR, yet this interpretation is the foundation of what justifies the development and marketing of medical food. In an exploratory workshop hosted by the National Academies of Sciences, Engineering, and Medicine, the FDA initiated some scientific dialogue among relevant stakeholders on what may constitute a DNR (10). That dialogue, however, focused largely on shifts in essential nutrient requirements (e.g., a shift in the Dietary Reference Intakes, a set of essential nutrient requirements published by the National Academies of Sciences, Engineering, and Medicine) as a direct result of a disease [10]. A more patient-centric approach, which considers the realities of patients managing not only a singular disease but also potential comorbidities, is essential to establish a definition that fosters research in the innovation of medical foods. The dietary management of a disease or condition requires the health care provider to consider a broad array of patient needs, including disease-induced nutrient requirements or nutrient requirements that are the cause of a disease, comorbidities that also alter nutritional requirements, mode of delivery of nutrition for the patient (i.e., orally fed, use of a feeding tube, or both), and of course, patient feasibility and sustainability to achieving an individuals’ nutrition care plan or Nutrition Care Process [11]. In other words, the health care practitioner's viewpoint of a distinctive nutritional requirement is specific to the nutrition care plan rather than simply the metabolic requirements of a specific nutrient.

Nutrition is a cornerstone of disease management. For healthy individuals, food can be readily and safely consumed. Conversely, people living with disease or other health conditions may be unable to orally consume food (or an adequate amount of food) to meet daily energy, protein, and essential nutrient needs or may have other medically determined nutritional requirements that go beyond what a normal diet can offer. In this context, medical foods can be designed not only to provide nutrients to patients who are unable to ingest and digest what is needed from the normal diet but also to provide an appropriate nutrient profile in an appropriate delivery mode that has been designed to improve nutritional status, health outcomes, and quality of life for people managing a disease or health condition.

In the example of inflammatory bowel disease, “managing the nutritional status of the patient can help to manage the episodes of disease” [2]. These patients are open to diet modifications and the use of medical foods, but they are challenged with insurance coverage for enteral therapies. Most insurers will only cover the cost when the product is administered through a nasogastric tube, even when patients prefer and can consume oral formulations [2,7,12].

The American Society for Parenteral and Enteral Nutrition Value Project elucidates another example of the need for specialized nutritional intervention in patient care. The findings of the American Society for Parenteral and Enteral Nutrition Value Project revealed that nutrition support could result in savings within the Medicare program of $580 million each year through shorter hospital stays and avoidance of complications [13]. The regulatory framework for medical foods must coincide with this need.

Further, more dialogue must be had on the interpretation of MODA as it relates to DNR. The narrow interpretation of DNR combined with MODA would limit the definition of medical food to a few physiologic conditions in which a product is necessary to sustain life. We refer to this as the “essentiality” principle. This principle is best illustrated by a classic example of a medical food: a product intended for the dietary management of an inborn error of metabolism. In these conditions, the patient has an enzyme defect that renders the patient unable to normally metabolize specific dietary components (e.g., in the case of phenylketonuria, patients cannot metabolize phenylalanine). The avoidance of the specific dietary component in these conditions is difficult to do through the normal diet, and if not done properly, the patient can experience significant adverse health impacts, such as severe permanent cognitive impairment and suboptimal nutritional status. To date, FDA has only explicitly cited inborn errors of metabolism (via its 2016 guidance) as meeting the criteria for a medical food.

Although HNC wholeheartedly agrees with the FDA’s recognition of these important products for inborn errors of metabolism as meeting the criteria of medical foods, a more modernized interpretation of DNR and MODA is needed to recognize the benefits of medical foods for other patients. The modernized interpretation should ensure that people living with disease have access to medical foods not only to live but also to live better.

### Interpretation of DNR: Where We Stand

HNC believes that a modernized interpretation of DNR can and should be adopted in light of putting the patient first and ensuring that nutrition is at the forefront of patient care. Various medical situations may require or benefit from the use of specialized medical food products to nutritionally manage different conditions. Given this thinking, our modernized interpretation of DNR is as shown in Table 1, which includes wording from FDA’s unimplemented 1996 ANPRM.

Furthermore, HNC believes abnormal physiologic manifestations or physical impairment would include the following conditions associated with acute and chronic disease or health conditions:

(i) A limited, impaired, or disturbed capacity to ingest, digest, absorb, metabolize, or excrete ordinary food or certain nutrients or metabolites or
(ii) Other medically determined requirements for nutrients or other food substances of biological value.

We ask that the FDA includes this language in statute to modernize and clarify the medical food category so that scientific advances can be developed into products to help the population for which medical foods were intended.

HNC considered 3 key pillars of nutrition care in developing this interpretation, as shown in Table 2. First, our interpretation of DNR would appropriately narrow the category of medical foods to products formulated with a high standard of scientific rigor and quality while also providing sufficient flexibility to address overall patient needs and support innovation and evolution in nutrition science. Although necessary adjustments to the intake of essential nutrients are an example of a DNR, it should not be the only accepted criterion for the classification of a product as a medical food. This restrictive interpretation creates a scenario in which products, such as standard enteral tube, feedings would be excluded from the medical food category despite their critical role in providing the sole source of nutrition to patients with distinctive physical impairments preventing them from consuming enough nutrition orally.

**TABLE 2:** Three key Pillars to a patient-centric approach to defining distinctive nutritional requirements

1. Focus on patients’ complete nutritional requirements beyond just nutrient levels. • Narrows the medical foods category to high-quality products formulated to a high standard of scientific rigor • Allows for sufficient flexibility to support innovation
2. Keep positive health outcomes for patients (through a focus on a reasonable means to achieve nutritional goals) as the core goal. • Places patients’ needs and quality of life first • Gives people living with disease the right to utilize medical foods to improve the quality of their medical care
3. Recognize that the continuum of disease severity can amplify the need for medical foods. • Recognizes that patients with severe disease may have additional comorbidities or be at risk of malnutrition • Prevents patients on the severe end of the disease spectrum from being precluded access to medical foods

Second, our interpretation of DNR is intended to be taken in a context in which the patient’s needs and quality of life come first, in contrast with the current interpretation of MODA that allows the development of medical foods only in situations of complete medical necessity. If it is impossible, impractical, unsafe, or nutritionally or clinically disadvantageous for people living with disease to meet their nutrition needs through diet alone, then they should be given the right to utilize medical foods to improve the quality of their nutritional care. In other words, the definition of DNR should not be limited to only those medical foods necessary to keep patients alive. The development of medical foods that support positive health outcomes is more consistent with population-based public health goals and nutrition guidelines.

Third, the severity of a disease or condition can vary along a spectrum, and patients with the most severe disease may be living with additional comorbidities that necessitate more complex nutritional modifications, may have or be at risk of malnutrition, and may require tube feeding. Although all patients with a disease or condition that necessitates nutritional modifications may benefit from the incorporation of a medical food into their Nutrition Care Process plan, it is important that the interpretation of DNR not be used to restrict the availability of disease-specific medical foods on the basis of the “healthiest” patient on the spectrum of severity. Patients on the severe end of the spectrum should not be precluded from having access to medical foods tailored to their needs because some patients living with the same disease or condition, with lesser severity, can successfully modify their normal diet with regular foods alone.

As HNC developed its definition of DNR, much of the discussion centered around the interpretation of “nutritional intake.” The term used in the medical food statutory definition is “distinctive ***nutritional*** requirement,” not “distinctive ***nutrient*** requirement.” This indicates the original intent of the category is broader than just an adjustment up or down or a narrower range of tolerance of only essential nutrients. Although these would certainly be examples of a DNR, they would not be exhaustive examples. DNR should incorporate a broader spectrum of types of nutritional and dietary modifications. Examples include, but should not be limited to, the following:

• Adjustments in the delivery of the food to enable nutritional intake (e.g., enteral tube feeding);
• Adjustments in nutritional intake, including nutrients that do and do not have a dietary reference intake (e.g., essential and conditionally essential macro- and micronutrients, amino acids, fatty acids, and/or other food substances of biological value);
• Adjustments, comparative to the healthy population, in the way one must monitor and continually adapt their nutritional intake (e.g., diabetes) and
• Adjustments of cellular energy metabolism.

HNC also considers that this definition of DNR should facilitate the development of products including but not limited to nutritionally complete formulas; nutritionally incomplete formulas, including individual “modular” type products (e.g., modular protein, carbohydrate, fat, fiber, vitamins, and minerals, or substances which belong to or are components of one of these); formulas for metabolic (genetic) disorders in patients aged >12 mo; and oral rehydration products. HNC also considers that tube feeds (specially formulated and processed products) should be medical foods. More specific examples of metabolic or physiologic and physical requirements that support the need for a modernized interpretation of DNR as it pertains to the medical food category were discussed at the Medical Foods Workshop [2] and are included in the Supplementary Text Appendix.

## Recommendations and Next Steps

### Adopt a modernized interpretation of DNR

Medical foods are an established solution widely used by health care professionals to manage the nutritional component of a patient’s disease state or condition. A more modernized interpretation of “distinctive nutritional requirements” is needed to match the evolution of nutrition science and the role of nutrition in patient care. The definition must be patient-focused and consider the spectrum of severity of disease, the patient’s quality of life, and the patient’s ability to sustainably maintain dietary modifications throughout their disease management. To accomplish this, we recommend implementing the definition and interpretation of DNR proposed here. We invite all stakeholders to consider emerging science to support patient needs and foster innovation in this field.

### Modernize the Medical Foods Regulatory Framework

To best reflect the evolution of nutrition science, health economics, and other health care research, the FDA should establish a modern interpretation of the regulatory framework for medical foods that allows room for innovation. Examples of entities that use a more modernized interpretation can be found domestically and globally. In 2016, coverage of medically necessary food and vitamins for certain conditions was included under the TRICARE program (sec. 714). The National Defense Authorization Act S. 2943 of December 2016 contained a provision (sec. 704) that would amend section 1077 of title 10, United States Code, “to provide TRICARE program coverage for medically necessary food, including the equipment and supplies necessary to administer that food and vitamins for digestive disorders and inherited metabolic disorders” [14]. By changing the coverage term *medical foods* to *medically necessary foods,* the TRICARE policy language (as in the National Defense Authorization Act noted above) modernized and expanded coverage to include nutritional innovations that were not captured by the regulatory interpretation of the definition of medical foods. This allows items not currently included in the narrow definition of medical foods to be included, such as medically necessary foods for digestive disorders. We include this clarification to serve as an example of how other entities and payers have modernized medical foods coverage as the science of nutrition has evolved.

Another example can be found in the European Union, where the defined need for medical food includes when it is impossible, impractical, unsafe, or nutritionally or clinically disadvantageous for the patient to satisfy their nutritional needs through the exclusive consumption of foods other than foods for special medical purposes (FSMP)—the term used in the European Union [9,15]. The European Commission’s recognition of nutritional and clinical advantage (as opposed to theoretical ability) to meet nutritional needs through the diet alone reflects a more modernized interpretation that considers patient nutritional needs. The ODA is currently too restrictive in recognizing the patient's benefit of nutrition care.

In addition, as explained during the 2017 introduction of the Medical Nutrition Equity Act: “The terminology **medically necessary foods** is being used instead of **medical foods** to avoid confusion with the ODA definition of medical foods. The ODA definition, which requires a product to be **specifically designed** to meet the **distinctive nutritional requirements** of a patient, is overly narrow in relation to how nutritional products are currently developed and used in the health care setting” [16]. The White House National Strategy, “Integrate Nutrition and Health,” reads as follows [17]:

“Prioritize the role of nutrition and food security in overall health—including disease prevention and management—and ensure that our health care system addresses the nutrition needs of all people.”

The role of medical foods in managing the dietary aspects of disease could be an important pillar of this work.

Previously, HNC responded to Docket No. FDA-2017-N-5094, titled “Review of Existing Center for Food Safety and Applied Nutrition Regulatory and Information Collection Requirements,” and provided FDA with recommendations to modernize the medical foods category [18]. These recommendations concerning the regulatory framework of medical foods included the following:

1) Repeal a portion of 21 CFR 101.9(j)(8)(ii), which states “...the dietary management of which cannot be achieved by the modification of the diet alone” [4]. This regulatory language is not supported by the statutory definition of medical foods and is being used to unnecessarily constrain the medical food category.
2) Revise a portion of 21 CFR 101.9(j)(8)(iii), which states, “…provides nutritional support specifically modified for the management of unique nutrient needs that result from the specific disease or condition, as determined by medical evaluation” [4]. (Note that FDA has used both *unique* and *distinctive* to describe the nutrient needs of patients who use medical foods.) HNC proposed to remove the words “unique nutrient needs that result from” so that the regulation would read “provides nutritional support specifically modified for the management of the specific disease or condition as determined by medical evaluation.” This would better reflect the physical and physiologic DNR of patients and eliminate confusion over “unique” nutrient needs.
3) Revise “Guidance for Industry: Frequently Asked Questions About Medical Foods, Second Edition” [6]. Currently, this guidance opines on certain diseases or conditions which fail to meet the statutory criterion of DNR without first defining DNR.

Nutrition science is moving faster than the regulatory framework, which is slowing the benefit to patients of innovation. The above recommendations call for continued research to provide FDA with the evidence needed to make the modernized regulatory framework a reality.

### Next Steps

Moving forward, HNC advocates the following next steps by medical foods stakeholders:

• Support ongoing nutrition research on the use and efficacy of medical foods, which allows room for innovation.
• Generate further evidence on the cost-effectiveness of nutritional care.
• Include patient advocates in the discussion.
• Propose suggested language for regulatory interpretation, as this paper set out to do.
• Encourage dialogue and collaboration with the FDA to explore how to address the messages shared here and in the proceedings of the Medical Foods Workshop.
• Advocate for necessary regulatory changes.
• Advocate for the role of medical foods in managing the dietary aspects of disease.

The acceleration of innovation in medical food research and the willingness of regulatory bodies to adapt to patient and health care needs as science advances will allow health care professionals and funding agencies to administer and reimburse these safe, cost-effective, and necessary products that are increasingly shown to improve patient’s quality of life while contributing to better health economic outcomes.

## Author contributions

The authors’ responsibilities were as follows – KC, BD, WJ, MJ, PP, JR: wrote the paper and had primary responsibility for the final content; and all authors: have read and approved the final manuscript.

## Conflict of interest

The authors report no conflicts of interest.

## Funding

The authors reported no funding received for this study.

## Appendix A Supplementary data

Multimedia component1 Multimedia component1

## References

[1] Medical Foods Workshop: Science, Regulation and Practical Aspects – Healthcare Nutrition Council. https://healthcarenutrition.org/medical-foods-workshop-2019/.

[2] Holmes J.L., Biella A., Morck T., Rostorfer J., Schneeman B. (2021). Medical foods: science, regulation, and practical aspects. Summary of a workshop. Curr. Dev. Nutr..

[3] 21 USC 360ee Grants and contracts for development of drugs for rare diseases and conditions. https://uscode.house.gov/view.xhtml?req=granuleid:USC-2000-title21-section360ee&num=0&edition=2000.

[4] CFR - Code of Federal Regulations Title 21. https://www.accessdata.fda.gov/scripts/cdrh/cfdocs/cfcfr/cfrsearch.cfm?fr=101.9.

[5] Regulation of Medical Foods (November 29, 1996). https://www.federalregister.gov/documents/1996/11/29/96-30441/regulation-of-medical-foods.

[6] Center for Food Safety and Applied Nutrition (October 31, 2022). https://www.fda.gov/regulatory-information/search-fda-guidance-documents/guidance-industry-frequently-asked-questions-about-medical-foods-second-edition.

[7] Berry S.A., Brown C.S., Greene C., Camp K.M., McDonough S., Bocchini J.A. (2020). Medical foods for inborn errors of metabolism: history, current status, and critical need. Pediatrics.

[8] Steele J. (March 1, 2016). https://easconsultinggroup.com/what-are-medical-foods-and-what-they-are-not/.

[9] Foods for special medical purposes/medical foods: A global regulatory synopsis. https://www.raps.org/news-and-articles/news-articles/2022/8/foods-for-special-medical-purposesmedical-foods-a.

[10] National Academies of Sciences (2018). https://doi:10.17226/25164.

[11] Nutrition Care Process. https://www.eatrightpro.org/practice/nutrition-care-process.

[12] Berry S.A., Kenney M.K., Harris K.B., Singh R.H., Cameron C.A., Kraszewski J.N. (2013). Insurance coverage of medical foods for treatment of inherited metabolic disorders. Genet. Med..

[13] Tyler R., Barrocas A., Guenter P., Torres K.A., Bechtold M.L., Chan L.-N. (2020). Value of nutrition support therapy: impact on clinical and economic outcomes in the United States. JPEN J. Parenter. Enteral Nutr..

[14] McCain J. (December 23, 2016). http://www.congress.gov/.

[15] European Commission (November 25, 2017). https://eur-lex.europa.eu/legal-content/EN/TXT/PDF/?uri=CELEX:52017XC1125(01).

[16] (February 7, 2017). Medical Nutrition Equality Act.

[17] The White House (September 27, 2022). https://www.whitehouse.gov/briefing-room/statements-releases/2022/09/27/executive-summary-biden-harris-administration-national-strategy-on-hunger-nutrition-and-health/.

[18] Public Comments – Healthcare Nutrition Council. https://healthcarenutrition.org/advocacy/public-comments/.

