# Supplementary material for: What is a “Distinctive Nutritional Requirement”? A Position Paper of the Healthcare Nutrition Council
Source: Curr Dev Nutr. 2023 Oct 7;7(11):102013. doi: 10.1016/j.cdnut.2023.102013 (PMC10637874; doi:10.1016/j.cdnut.2023.102013)
Supplement: Multimedia component1 [file mmc1.docx]

**SUPPLEMENTARY TEXT APPENDIX**

**Title:**

**What is a “Distinctive Nutritional Requirement”? A Position Paper of the Healthcare Nutrition Council**

**First Author:**

**Berit Dockter MPP, RD, LD**

**Foods for Special Dietary Use**

Another category that is useful to compare with that of medical foods is FSDU. Per regulation,^1^ “the term special dietary uses, as applied to food for man, means particular (as distinguished from general) uses of food, as follows:

1. Uses for *supplying particular dietary needs* which exist by reason of a physical, physiological, pathological or other condition, including but not limited to the *conditions of diseases*, convalescence, pregnancy, lactation, allergic hypersensitivity to food, underweight, and overweight;
2. Uses for supplying particular dietary needs which exist by *reason of age*, including but not limited to the ages of infancy and childhood;
3. Uses for supplementing or fortifying the ordinary or usual diet with any vitamin, mineral, or other dietary property. Any such particular use of a food is a special dietary use, regardless of whether such food also purports to be or is represented for general use.” (21 CFR 105.3(a)(1)(i)).

HNC interprets this as a category of products used to play a role in disease-compatible diets, for the everyday health of a person living with a disease to provide benefits to the quality of life of someone living with disease. Examples such as sugar-free pancake syrup work well within the dietary restrictions of a disease or condition. Whereas medical foods are products clearly defined to manage a disease or condition, the interpretation of FSDU has often been less clear, and FDA has not opined on their interpretation of the category and special dietary needs for a disease. FSDU must follow nutrition labeling regulations in 21 CFR 101.9. While these therapeutic food categories sound similar, the key difference is the “distinctive nutritional requirements” definition (based on recognized scientific principles, established by medical evaluation…) for medical foods versus the “special dietary need” definition for FSDU. Medical foods, additionally, should be used under medical supervision, and FSDU are not required to be used under medical supervision but could be part of a health care professional’s recommendation for the patients’ diet and adherence to nutritional recommendations. Understanding the basics of the FSDU category provides context for HNC’s definition of “distinctive nutritional requirements.” The proposed definition encompasses both physiological nutrient changes but also physical impairments that result in a change to nutritional management.

**Other Support for a Modernized Interpretation of DNR**

More specific examples of metabolic or physiological and physical requirements supporting the need for a modernized interpretation of DNR as it pertains to the medical food category are discussed below.

*Metabolic or Physiological Requirements*

Inborn errors of metabolism are examples of metabolic conditions that prevent individuals from metabolizing nutrients in a normal way, necessitating the need for medical foods to meet the nutritional needs of the individual to prevent clinical complications and sustain life. For example, phenylketonuria (PKU) is a condition, occurring in 1 in 10,000 to 15,000 infants in the United States, that prohibits the individual from being able to metabolize phenylalanine, thus causing phenylalanine to accumulate in the body at harmful levels.^2^ Maple syrup urine disease (MSUD) is another inborn error of metabolism occurring in less than 1 in 185,000 infants worldwide.^3^ In this condition, branched amino acids and the corresponding keto acids accumulate in the blood and can cause life-threatening seizures, coma, and brain damage.^3^

In both these conditions, it is impractical for individuals to modify their diets to carefully control their intake of the offending amino acid(s) while still meeting their nutritional needs, without the use of medical foods. Medical foods provide the nutrition needed to support growth and development while managing the symptoms of their condition. PKU and MSUD are just two of many inborn errors of metabolism which require the use and consumption of medical foods.^4^

Medical foods can also be formulated to support improved patient health and quality of life for other physiologically determined DNRs that result from diseases or health conditions. For example, as renal patients lose the ability to regulate electrolytes, they require a reduced intake of potassium and other nutrients.^5^ It may be difficult for these patients to maintain adequate protein and electrolyte status with conventional foods or diets alone. As end-stage renal disease progresses, the patient may need to consume more and more of their daily nutrient intake using medical foods. Medical foods, in this case, can effectively allow for the dietary management of the DNR of this medical condition.

An illustration of cost efficiency and improved health outcomes with medical nutrition is found with Crohn’s disease. An estimated 780,000 people in the United States have Crohn’s disease. The cost to provide medically necessary food for dietary management is only about $10 per day;^6^ however, the cost to provide drug therapy in the form of biologics to induce remission may be over $55 per day.^7,8^ We argue that a narrow interpretation of medical foods and DNR (as an adjustment to the nutrient level required alone) prevents medical foods from being utilized and covered by payers for patients with Crohn’s disease both because of the absence of a specific DNR and because many of these patients consume their enteral nutrition orally. Studies have shown the effective use of exclusive enteral nutrition.^9–11^ Therefore, inclusion of these products in a modernized interpretation of the medical foods framework would benefit the health system.

In the acute care setting, the role of specific conditionally essential nutrients has been researched for several decades. Both surgical and trauma patients have been shown to have increased risk of infections in part due to the depletion of arginine, an amino acid that becomes conditionally essential in these states of stress.^12^ Clinical trials have shown reduced rates of infections, improved surgical site healing, and reduced length of stay with supplementation of arginine for some major elective surgeries.^12^ Medical foods can provide a means to clinically improve nutritional status through a reasonable provision of arginine and other necessary nutrients.

In summary, the use of medical foods for conditions in which the patient cannot reasonably achieve required nutrients by MODA provides these patients with immense benefits.

*Physical Requirements*

Patients who are unable to orally ingest sufficient nutrition require nutrition support by enteral tube feeding products or texture-modified foods and liquids. Enteral tube feeding products are available with a wide range of nutrient profiles. This enables the health care practitioner to provide tailored medical nutrition therapy that best supports the patient, whether it be through a disease-specific enteral feeding, or, in some cases, an immune-modulating enteral feeding that better prepares the patient to withstand the rigors of a major operation or for recovery after surgery, trauma, or a medical treatment. For the situations in which consuming conventional foods to meet the modified nutritional needs during recovery is not possible, medical foods that meet both a physical and a physiological need allow the patient to function at a higher level than would otherwise be possible.

As one example, in cystic fibrosis patients, nocturnal enteral nutrition has been shown to improve nutritional status and lung function in moderately malnourished patients.^13^ The use of nocturnal enteral nutrition tube feeding for cystic fibrosis patients ensures these patients will achieve their required nutrient intake, which can improve nutritional status outcomes.^13^

**References**

1. CFR - Code of Federal Regulations Title 21. Accessed December 8, 2022. https://www.accessdata.fda.gov/scripts/cdrh/cfdocs/cfcfr/cfrsearch.cfm?fr=101.9

2. Phenylketonuria: MedlinePlus Genetics. Accessed December 8, 2022. https://medlineplus.gov/genetics/condition/phenylketonuria/

3. Maple syrup urine disease: MedlinePlus Genetics. Accessed December 8, 2022. https://medlineplus.gov/genetics/condition/maple-syrup-urine-disease/

4. Berry SA, Brown CS, Greene C, et al. Medical Foods for Inborn Errors of Metabolism: History, Current Status, and Critical Need. *Pediatrics*. 2020;145(3):e20192261. doi:10.1542/peds.2019-2261

5. Potassium and Your CKD Diet. National Kidney Foundation. Published January 7, 2016. Accessed December 13, 2022. https://www.kidney.org/atoz/content/potassium

6. Crohn’s and Colitis Foundation. Co-Sponsor HR 3783/S 2013, the Medical Nutrition Equity Act. Published online 2021. https://www.crohnscolitisfoundation.org/sites/default/files/2021-08/MNEA%20-%20August%202021.pdf

7. Canadian Agency for Drugs and Technologies in Health; 2016. Table 1, Cost Comparison Table of Biologics for the Treatment of Crohn’s Disease. Published December 2016. Accessed December 8, 2022. https://www.ncbi.nlm.nih.gov/books/NBK424364/table/T68/

8. How coverage of medical foods can save lives & costs. Patients and Providers for Medical Nutrition Equity. Accessed December 9, 2022. https://www.npkua.org/Portals/0/pdfs/PPMNE-MNEA_factsheet_iem.pdf

9. Borrelli O, Cordischi L, Cirulli M, et al. Polymeric diet alone versus corticosteroids in the treatment of active pediatric Crohn’s disease: a randomized controlled open-label trial. *Clin Gastroenterol Hepatol*. 2006;4(6):744-753. doi:10.1016/j.cgh.2006.03.010

10. Penagini F, Dilillo D, Borsani B, et al. Nutrition in Pediatric Inflammatory Bowel Disease: From Etiology to Treatment. A Systematic Review. *Nutrients*. 2016;8(6):334. doi:10.3390/nu8060334

11. Lee D, Baldassano RN, Otley AR, et al. Comparative Effectiveness of Nutritional and Biological Therapy in North American Children with Active Crohn’s Disease. *Inflammatory Bowel Diseases*. 2015;21(8):1786-1793. doi:10.1097/MIB.0000000000000426

12. Rosenthal MD, Carrott PW, Patel J, Kiraly L, Martindale RG. Parenteral or Enteral Arginine Supplementation Safety and Efficacy. *The Journal of Nutrition*. 2016;146(12):2594S-2600S. doi:10.3945/jn.115.228544

13. Steinkamp G, von der Hardt H. Improvement of nutritional status and lung function after long-term nocturnal gastrostomy feedings in cystic fibrosis. *J Pediatr*. 1994;124(2):244-249. doi:10.1016/s0022-3476(94)70312-4
